# Contributions of anatomy to forensic sex estimation: focus on head and neck bones

**DOI:** 10.1080/20961790.2021.1889136

**Published:** 2021-07-01

**Authors:** Thamires Mello-Gentil, Vanessa Souza-Mello

**Affiliations:** Department of Anatomy, Rio de Janeiro State University, Rio de Janeiro, Brazil

**Keywords:** Forensic sciences, forensic anthropology, sex estimation, anatomy, head, neck, teeth

## Abstract

This study sought to provide an up-to-date review of the importance of anatomy to human identification, focusing on the usefulness of anatomical knowledge about the head and neck bones and teeth to sex estimation in routine forensic anthropology methods. A detailed search of osteology applications in forensic sex estimation was conducted through the electronic databases for the 10 years prior to July 2020. Relevant articles and classic literature on the subject were gathered and are outlined in this review. Among the available literature, several metric analyses showed accuracy superior to 80% in sexual diagnosis. Angles measured from the inclination of glabellae and analysis of the external frontal bone surface through three-dimensional computer-aided design emerge as reliable cranial indexes for sex estimation. In the mandible, the condylar and coronoid height, bigonial width, and coronion–gonion distance express significant sexual dimorphism. Measurements of the canine are the best option for sex estimation using teeth, as well as the thickness of the dentine or enamel of incisors. The axis vertebra surpasses other neck bones for sex estimation due to its atypical shape and the presence of the odontoid process. Metric analyses based on anatomy can provide reliable accuracy in sexual diagnosis. Adequate training and anatomical knowledge can reduce bias and interobserver differences, and the use of three-dimensional models and computed tomography images can enhance the accuracy of these methods for sex estimation. However, every method should be validated before being applied to a different population.

**Key Points**
• Anatomy-based metric analyses can provide reliable accuracy in forensic sex estimation.

• Glabellae inclination, external frontal bone surface, mandible, and canine teeth measurements can reach accuracies superior to 80% in sexual diagnosis.
• The use of three-dimensional models and computed tomography images can enhance accuracy in sex estimation.
• Every method should be validated before being applied to a different population.

## Introduction

The head and neck are vital parts of the human body, although they represent a small part of the body’s surface [1]. The bones of the head and neck are mainly derived from the branchial arches and start to develop in the fourth week of pregnancy [2]. Anatomically, the head comprises 22 bones, and the neck has seven cervical vertebrae and the hyoid bone [3–5]. The maxilla and mandible are also related to the superior and inferior dental arches, in which odontogenesis occurs in different age ranges for the deciduous and permanent dentitions [6].

The human skeleton is completely renewed every 10 years of postnatal life. Around 3%–10% of human bones are renewed each year, highlighting the fact that bone tissue is highly dynamic and undergoes continuous remodelling throughout life [7,8]. Bone remodelling is linked to calcium homeostasis and involves the synthesis and release of many factors into the bloodstream, making this tissue more active than its previous strictly structural concept [9]. This important remodelling feature allows skull bones to be relevant aid structures in the human identification process [10]. In this context, parts of the skeleton of the head and neck are essential factors for sexual dimorphism [11,12]. Thus, anatomical knowledge applied to anthropological studies can assist with sex estimation, the first step in human identification.

Forensic anthropology involves multidisciplinary work, including adequate anatomical knowledge, which can provide correct human identification through low-cost methods [13]. Anatomical know­ledge is crucial in cases such as mass disasters, na­tural disasters, carbonised bodies, and bodies in an advanced stage of decomposition, especially when skeletal remains are the only pieces available for identification [14,15]. In view of their forensic functionality, the bones of the head and neck and the teeth emerge as fundamental structures, considering their resistance to high temperatures, especially the teeth [16,17]. This review sought to discuss the associations between anatomical knowledge and human identification, highlighting the usefulness of the anatomy of the head and neck bones and the teeth to sex estimation in the routine methods of forensic anthropology.

## Anatomical and embryological basis: head and neck bones

The anatomy of the bones of the head and neck, and the embryology of these structures, are complex, with peculiar characteristics at different stages of life, justifying their importance for surgical approaches and human identification processes. The head bones comprise the skull, studied in two parts: the neurocranium (surrounds and protects the brain, formed by eight bones in adults) and the viscerocranium (the facial skeleton — includes the orbit, the beginning of the digestive tract and the respiratory tract, composed of 14 bones). The bone structures of the neck comprise seven cervical vertebrae (protect the spinal cord in this segment) and the hyoid bone (important for coordination of swallowing and breathing) [2,4].

Embryologically, the skull originates from the mesoderm, and the neural crest and its components will fuse firmly throughout life, undergoing continu­ous remodelling from pregnancy to adulthood. The development of the skull occurs in the embryonic period (1–8 weeks), while ossification begins in the foetal phase (7 weeks onwards). The branchial or pharyngeal arches are structures that give rise to the bones of the skull, muscles, nerves, and associated blood vessels. They are formed from the 19th to 21st days of gestation and are in five pairs in the human species [2,4]. The bones of the head and neck most frequently applied to routine forensic anthropology will have their embryology and anato­my detailed below.

The frontal bone stems from the cells of the neural crest, as do other bones of the anterior region of the skull (ethmoid, sphenoid and squamous part of the temporal bones, and the viscerocranium). It forms the roof of the orbit and connects to the zygomatic bone to constitute the orbit’s lateral margin. It also forms the posterior aspect of the orbit when articulating with the lacrimal, sphenoid, and zygomatic bones, and articulates with the maxilla and the greater wing of the sphenoid bone in the anterior cranial fossa, and the nasal and the parietal bones [1,18]. It is a unique bone in adulthood, after the disappea­rance of the metopic suture between 3 and 19 months of age [19], a process that can extend up to 8 years of age [1]. Conversely, the persistence of the metopic suture in adulthood, named metopism, is considered an anatomical variation and occurs in around 5% of the population [20].

The frontal bone is classified as a pneumatic bone because it has two cavities inside it (the frontal sinuses) in adults. These structures are filled with air and have a triangular shape. They derive from the migration of ethmoidal air cells to the area between the internal bone plate and the external bone plate in the 16th intrauterine week. They are rudimentary at birth, begin to develop between 1 and 2 years of life, reach a considerable size in childhood (7–8 years), but only reach maturation in early adulthood [21,22]. They present frequent anatomical variation in terms of shape and development, in addition to pronounced sexual dimorphism [23,24].

The mandible is a major bone of the skull and comprises a vertical ramus and a horizontal body, which are connected by the gonial angle, whose midpoint is known as the gonion. The ramus is related to muscles and ligaments that are important for chewing, whereas the upper border of the body of the mandible has alveolar processes, related to the lower teeth. Its formation begins in the 6th week of intrauterine development when the first branchial arch originates Meckel’s cartilage. This cartilage indicates the future localisation of the mandible but does not form it. The mandible ossifies from two ossification centres by intramembranous ossification. At birth, there are two mandibles, which merge at the mandibular symphysis throughout the first year of life [25,26].

The eruption of the deciduous teeth follows mandibular ossification. The primary teeth (20) and the permanent teeth (32) stem from ectoderm and ectomesenchyme, cells of the neural crest that migrate to the oral cavity. Odontogenesis begins between the 6th and 8th weeks of intrauterine development for the primary dentition and in the 16th week for the permanent dentition. The enamel is derived from the ectoderm, whereas the other elements (dentine, cementum, periodontal ligament, and dental pulp) derive from the ectomesenchyme. The mandibular central incisors are usually the first to erupt in the primary dentition, between 6 and 10 months of age, whereas the third molars are the last of the permanent dentition to erupt, between 17 and 21 years of age [6,27,28].

In the neck, the seven cervical vertebrae arise from the somites, which form the sclerotome. During the 4th week of embryogenesis, the sclerotome deve­lops rapidly around the notochord and the neural crest, initiating the spine formation process. The cervical vertebrae appear as independent structures in the 6th week of embryonic development when the ossification process begins with three main centres of ossification. During the first year of life, two secondary ossification centres appear [29,30].

Ossification of the axis dens or odontoid process takes longer, and misdiagnoses of fractures in this location often occur in paediatric patients. Complete fusion of the primary ossification centre of the axis dens with the vertebral body of the axis occurs at the age of 6 years, whereas the secondary ossification centre — the terminal ossicle at the tip of the dens — ossifies at around the age of 12 years. The atypical atlas and axis vertebrae are mostly used in the human identification process as they (and C7) are different from the typical vertebrae (C3–C6) because they do not have all the following characteristics: rectangular vertebral body, triangular vertebral canal, foramen of the transverse process and bifid spinous process [1,31,32].

## Forensic anthropology in human identification

According to the International Criminal Police Organization (INTERPOL)’s Disaster Victim Identification Guide (2018), human identification comprises primary methods (fingerprint, dental, and DNA analyses) and secondary methods (recognition, tattoos, anthropology, and others), which are used to compare antemortem and postmortem data [33]. Among the primary methods, dental analysis is of paramount importance, mainly because the teeth have substantial resistance to high temperatures and very particular characteristics. Dental analysis is essential in cases of identification of burned and skeletonised bodies. DNA is a resource that has been increasing its security rate in proving results. However, it is not yet a fully consolidated method, in addition to being high cost [34].

Forensic anthropology stands out among the se­condary methods. Hence, among other attributions, anthropological knowledge employs its methods and techniques for legal, civil, and humanitarian purposes [35]. Forensic anthropologists should be well trained in osteology to deal with the identification of skeletal remains, regardless of whether they are skeletonised, decomposed, burnt or dismembered [36]. It is of considerable relevance because it is the area of science that applies the study of humans from a cultural and physical point of view.

Physical anthropology relies on the morphological and metric aspects of bone structures to estimate and determine the main characteristics of an individual. Thus, bones are structures of great value in identification because they assist in estimating sex, species, ethnicity, height, and age. A thorough knowledge of osteology can guide successful and reliable human identification. A recent study showed that osteology provides reliability in sex estimation if the bone is well preserved and belongs to an adult, and that proteomics can enhance the reliabili­ty of the results when combined with osteology [37]. Thus, in-depth study of the bone structures that contribute to a positive result in the identification process is essential. Because the head and neck region is an area that covers important bones, the following section will detail the contribution of these anatomical structures to sex estimation [35,38].

## Sex estimation

The estimation of sex is the first step in human identification, mainly because the estimation of other elements (age, ancestry, and height) follows patterns related to sex [39,40]. The pelvic bone expresses the most significant sexual dimorphism, followed by the bones of the skull. However, the cervical vertebrae and the teeth have been increasingly studied for sex estimation because large bones can be recovered fragmented, suffer more postmortem changes, and are more prone to damage in mass accidents or calcination in fire cases [41,42].

It is important to bear in mind that sexual dimorphism becomes more apparent after puberty because of bone changes induced by sexual hormones, albeit with around 10% of sexual dimorphism relating to bone size [36]. Hence, pure observational morphoscopic studies rely on the expertise of the anthropo­logist and can show high interobserver error (<10% is acceptable) [43]. Moreover, most methods described in the literature apply to the adult skeleton; sex estimation in child or juvenile bones is still a huge challenge. In juveniles, accurate results (accuracy > 80% is acceptable) come from genomic methods [37].

Methods that combine osteology with metric analysis increase confidence in the results, eliminating the subjectivity sometimes attributed to forensic anthropology [44]. Morphometric methods rely on the expertise of the anthropologist in observing the details of bone landmarks, followed by measurements that can help to construct discriminant functions to estimate sex. These methods can reach acceptable accuracy (>80%) in sex estimation, and are valuable [43,45]. However, they must be often updated and validated before being applied to different populations because a miscegenated population can reach 30% similarity between bone measurements of men and women [46]. A disadvantage of morphometric methods is that the manipulation can cause secondary damage to the bone [47].

More recently, computer-aided measurements have brought technology to routine forensics. Nowadays, forensic identification can benefit from geometric morphometrics or even three-dimensional (3 D) reconstruction of bone landmarks or structures from computed tomography (CT) images or magnetic ­re­sonance imaging (MRI) to apply quantitative methods with high accuracy. CT and MRI offer useful data on anatomical structures without compromising spatial interrelation, and are increasingly used in forensic cases [48,49]. However, these methods also depend on the ability of the observer to perform the protocol to avoid interobserver error > 10%.

### Skull

In the skull, the upper third of the face expresses substantial sexual dimorphism. The frontal bone contributes to these findings with landmarks, craniometric points, the external surface, and the frontal sinuses. These structures provide significant accuracy (over 80%) in estimating sex from geometric morphometric measurements from 3 D virtual models or analysis of CT images or radiographs [13,43,50].

#### Frontal bone

**Figure 1.**
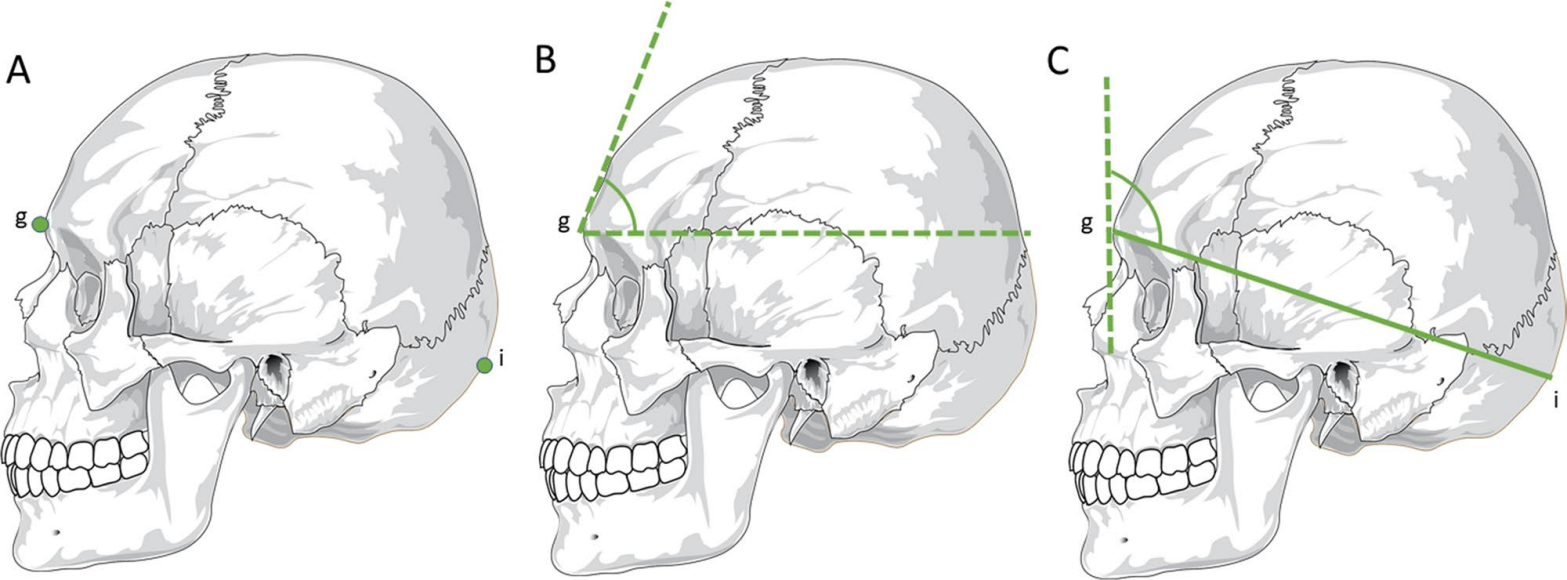
Metric analyses with statistical significance in sex estimation — skull. (A) Craniometric points — glabellae (g) and inion (i). (B) Glabellae inclination angle — a line is drawn from the glabellae parallel to the Frankfurt plane and another line tangential to the frontal bone. The resulting angle is smaller in men than in women. (C) Schwalbe’s frontal angle — a horizontal line is drawn connecting the glabellae to the inion and a vertical line tangential to the most prominent point of the frontal bone in the median sagittal plane.

Male skulls exhibit a more inclined forehead, along with more prominent glabellae and supraciliary arches than female skulls. In contrast, female crania show a more vertical forehead coupled with a more pronounced frontal eminence [1,51]. The use of the frontal bone in the sexual diagnosis process encompasses the analysis of its inclination to the glabellae [52–54]. To this end, the glabellae (Figure 1A) is defined as the most prominent point in the glabellar region (between the two superciliary arches), which is more voluminous in male skulls. A line is drawn from the glabellae parallel to the Frankfurt plane and another tangential to the frontal bone, and the resulting angle is the inclination of the frontal bone, which is smaller in males than females (Figure 1B). A cut-off value of 78.2° for glabellar inclination angle separates male (under this value) and female individuals (above this value) and the accuracy was 75%–82% in the North American and Portuguese populations. However, in the Chinese population, the accuracy was only 66%, which is not satisfactory [53]. In the Athens collection, the glabellae–frontal bone profile correctly classified male skulls in 82.4% of cases and female skulls in 87.8% of cases. In this study, the percentage of correct classification increased when conjugated with the height of the mastoid process and the profile of the occipital bone [54].

More recently, among 10 different angles evaluated based on different craniometric points, the most reliable in sexual diagnosis was the Schwalbe’s frontal angle (Figure 1C). This is the angle formed by a vertical line tangential to the most prominent point of the frontal bone in the median sagittal plane, and a horizontal line connecting the glabellae to the inion (the most pronounced point of the external occipital protuberance, Figure 1A). In this study, there was an accuracy of 75.4%, and the discriminatory value was 88.6° in the Caucasian population, with values above this point suggestive of female skulls with more vertical foreheads, and lower values suggestive of males with more inclined foreheads [52].

The analysis of the volume of the glabellar region and the superciliary arch, using 3 D models, revealed a significant statistical difference between the sexes in early Californian Indians (*P* = 0.0083) and modern Portuguese crania (*P* = 0.0051). This measure surpassed other sites such as the nuchal line, mastoid process, and supraorbital margin in these populations, although it did not provide a statistically significant difference between males and females in modern African-Americans and late California Indians [55]. Similarly, a Colombian study confirmed that differences in the size and shape of the glabellar region are useful tools for sexual diagnosis based on quantitative morphometric and geometric data. It showed an accuracy of 84.31% using a discriminant analysis based on four landmarks from lateral radiographs of the glabellar region [56].

Three-dimensional analysis using CT revealed 95.5% accuracy in sex estimation with a linear discriminant function with eight endo- and ectocranial variables. It reached 97.3% accuracy when using 11 variables. Once again, the glabellae (glabellar projection index) showed an accuracy of 82.4% when assessed separately, reinforcing the importance of this bone landmark in estimating sex [57].

An Australian study using 3 D analysis of 18 measurements among 31 bone landmarks in the skull confirmed the importance of establishing specific standards for each population, reaching 90% accuracy and 2.1% sexual bias [58]. Similarly, a model of nine variables, based on 27 distances between bone landmarks and craniometric points of CT scan images of Tunisians, reached 90% accuracy and 2.9% bias in estimating sex [59]. These methods, when applied to other countries, resulted in significant bias in sex estimation (mainly female skulls) and an accuracy of less than 80%.

The external surface of the frontal bone has also emerged as a relevant tool for sex estimation, with considerably different results depending on the popu­lation studied. In this way, the roundness of male and female frontal bones was assessed by aligning a 3 D CT model of male and female frontal bones with a 3 D computer-aided design sphere in Turkey. This approach revealed a predominance of spherical shapes in men over women in a Turkish sample, with 77.5% accuracy, questioning the roundness commonly attributed to female foreheads in other populations [51]. Conversely, in the Czech population, this same method reached 72.8% in sex estimation, 5% lower than the success rate in the Turkish population [60].

Among Czech people, evaluation of the form and shape of the external frontal surface, using 3 D virtual models from CT images, achieved 83.49% accuracy in sex estimation after cross-validation. Of note, the addition of the frontal bone volume did not improve the rate of success, emphasising the importance of the frontal bone external surface when it comes to sexual diagnosis in this specific population [13].

In the Chinese population, the use of the superior orbital margin and the frontal bone surface in a more objective method involving wavelet transform and Fourier transform reached 90.9% and 94.4% in sex estimation for males and females [47]. Additionally, the diploe thickness, which is more pronounced in men and older individuals, can assist with sex estimation in the skulls of elderly people [61]. These methods that involve the robusticity of bone landmarks can display more noticeable sex dimorphism in males aged over 30 years and women aged over 45 years [62].

#### Frontal sinuses

The frontal sinuses complete their formation at around 20 years of age and are structures that, like fingerprints, present uniqueness, differing even between homozygous twins [63]. Therefore, they can be used for human identification, especially concerning sex estimation, albeit with some limitations.

A radiomorphometric analysis of the frontal sinuses in the Indian population achieved 61% accuracy in estimating sex, which is not a satisfactory index [64]. However, the combination of the dimensions of the frontal sinuses (width and height, which tend to be large in men) with nasal septum patterns from radiographs can be more reliable in estimating sex [65]. These studies used small samples (80–100 individuals), which may have influenced the results.

In the Chinese population, the use of the frontal sinus index and frontal sinus area, calculated from lateral cephalometry of 475 patients in a discriminating function resulted in an accuracy of 76.6% in the identification of sex [66]. However, the use of posteroanterior radiographs of 300 Indians for the analysis of logistic regression showed an accuracy of only 64.6% in estimating sex [67].

The use of CT with linear and volumetric measurements of the frontal sinus in the three anatomi­cal planes led to an accuracy of 66.5% in estimating sex in the Brazilian population [68]. Cone-beam computed tomography (CBCT) resulted in 80% accuracy in sexual diagnosis using the frontal sinus [69] and 92%–96% when the frontal sinuses were evaluated together with the maxillary and sphenoid sinuses [70]. The simultaneous use of the external frontal bone surface coupled with the frontal sinus surface and volume in virtual 3 D models from CT images resulted in 98.05% accuracy in estimating sex in the population of the Czech Republic, reaching 84.46% accuracy after cross-validation [13].

The low accuracy of the frontal sinus for the estimation of sex stems from the substantial inter-individual variation. However, this structure must be considered in forensic analysis when other more robust methods for sex estimation are not available [71].

#### Mandible

Pelvic and skull bones are the pioneering choice for sex estimation because of the high degree of relia­bility in the result, reaching 97% accuracy when analysed together [38]. However, in the absence of these first-choice structures, the mandible is the best option. Bone remnants and isolated structures are the most common findings in forensic practice, and the mandible, considered the most resistant bone of the face, when found intact, is capable of providing a high rate of identification effectiveness [72,73].

The mandible is a unique and mobile bone that, in addition to housing the lower dental arch, connects with the skull through the temporomandibular joint. The use of this bone structure for sexual estimation occurs through qualitative and quantitative analysis. In general, when it comes to the qualitative aspect, male mandibles have a lower degree of deli­cacy, with more robust anatomical characteristics. The male mandible has a rougher structure because of its more pronounced muscular insertions and the greater masticatory forces exerted in men than in women [74,75].

Quantitative analysis uses metric aspects to support the study results. In addition to male characteristics being more prominent, metric values from different mandibular measurements stand out when compared with female values. Despite research carried out in different geographic regions, which indicates different ethnicities, there is variation in the measurements found, but with few exceptions, the numerical results of females are lower than those of males [72,74,76]. In modern humans, the female mandible measures 92.4% of the size of the male mandible, confirming its importance in sexual diagnosis [77]. Still, it is necessary to develop standards of sexual estimation with accuracy for each population [72].

The mandibular ramus and the condyle have been widely used as indicators for sexual diagnosis because they are sites of extensive morphological change that contribute to sexual dimorphism [77]. The shape of the mandibular ramus reached 92.1% accuracy in estimating sex in white American women and 93.8% accuracy in Amerind American men [78]. Ramus flexure showed 99% reliability in sex estimation in Africans [78], whereas the maximum mandibular length exhibited the highest accuracy (81.8%) in ­se­xual diagnosis among 11 metric parameters evaluated in CBCT images from Chinese mandibles [79].

The condylar process, located at the upper posterior end of the ramus, is the target of several anthropometric studies due to its bone remodelling characteristics [80]. In mandibles from the Northern Indian population, the indicators with high accuracy in sexual diagnosis were coronoid height (74.1%) and condylar height (72.4%), when evaluated alone. However, a five-parameter discriminant function resulted in 80.2% accuracy in the same sample, with the female sex having the highest degree of fidelity in the results of the estimation [72].

**Figure 2.**
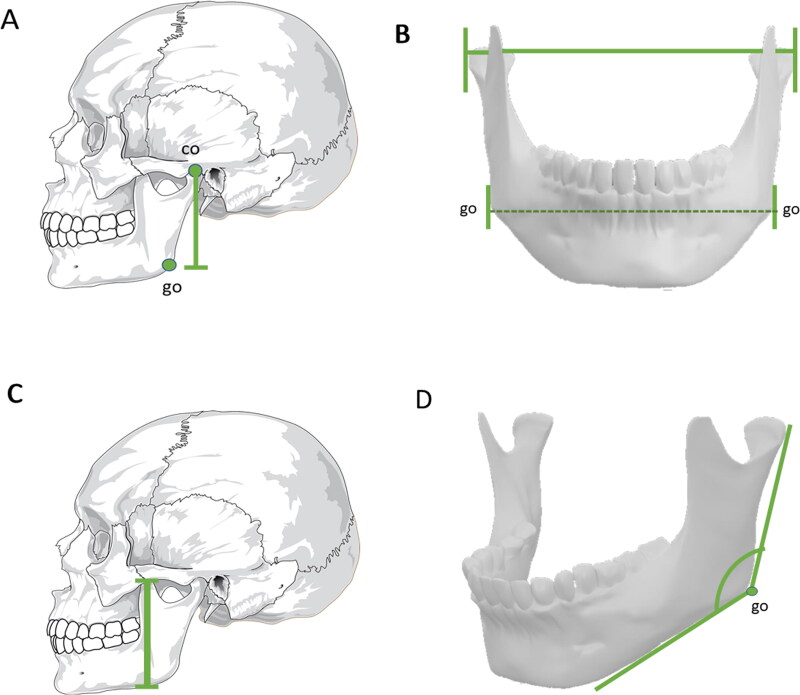
Metric analyses with statistical significance in sex estimation — mandible. (A) Coronion (co) — gonion (go) distance. (B) Bigonial width and bicondylar distance. (C) Mandibular length. (D) Gonial angle. These measurements exhibit greater values in males, whereas the gonial angle is usually smaller in males than in females.

A study carried out on adult mandibles in Brazil showed that the measure with the greatest evidence of dimorphism was the coronion–gonion distance (Figure 2A), with the male metric average being 13.40% greater than that of the female [81]. In mandibles from south Indian adults, bigonial breadth (*P* < 0.0001, Figure 2B), bicondylar breadth (*P* < 0.0001, Figure 2B), and mandibular length (*P* < 0.0001, Figure 2C) were shown to be statistically significant for sexual dimorphism, with lower values in the female measurements [76]. Additionally, the posterior border of the mandibular ramus varies in relation to the occlusal level. In most cases, the female mandibular ramus without a straight line indicates a more youthful shape [78].

The gonial angle is formed by two straight lines that leave the gonion point, one towards the most posterior point of the condyle and the other towards the chin (Figure 2D). The gonial angle emerges as a valuable tool to estimate sex, but not age [82]. Studies carried out in Portuguese [83] and Han Chinese populations [79] proved that gonial angular measurements on the left side presented higher values in adults of both sexes than the right side, whereas the left and right gonial angles exhibited lower values in males than in females, showing its significance in sexual diagnosis, with 73.5% accuracy among the Chineses. However, this measurement showed only 61.1% accuracy in the Portuguese population [83]. Although the gonial angle alone represented the lowest accuracy to estimate sex in Brazilian people [84], the use of four variables (ramus length, gonial angle, bicondylar breadth, and bigonial breadth) from CBCT scans to construct a discriminant function reached 95.1% accuracy in sex estimation in this highly miscegenated population [85]. In Egyptians, the use of discriminant analysis with images from spiral CT showed that the gonial angle was among the three most accurate mandibular measurements (>83%) to estimate sex [86].

#### Dental morphology

The dental arch is an important tool in the human identification process. Teeth are structures of great value for forensic medicine because they have a resistant chemical composition, in addition to mechanical and thermal characteristics that preserve the dental organ in the postmortem degradation processes [87,88].

Dental morphometry allows analysis that is significant in sexual dimorphism [89]. Crown and root measurements, mesiodistal and buccolingual distances, and tissue and morphological characteristics of the teeth have been relevant to confirm the potential for sexual estimation [88,90,91]. The permanent dentition presents itself as the most sexually dimorphic, and the best samples come from teeth in the early permanent dentition because they show less wear than older teeth [91–93].

In modern populations, studies confirm that male measurements surpass female ones [87,88,91,94]. Nevertheless, the accuracy of metric analyses of the canines is higher in females, ranging from 78.9% to 100%, than in males, ranging from 76.2% to 90.5% [91]. Canine teeth, when compared with other teeth in both arches, are of great interest in sexual estimation because of their substantial resistance to trauma and periodontal damage, as well as exhibiting a significant measure difference between the sexes [87,91,95]. However, the values vary in different populations [96]. An additional explanation for the sexual dimorphism of the canine tooth is its evolutionary origin, given that male primates used this tooth in hunting [97].

**Figure 3.**
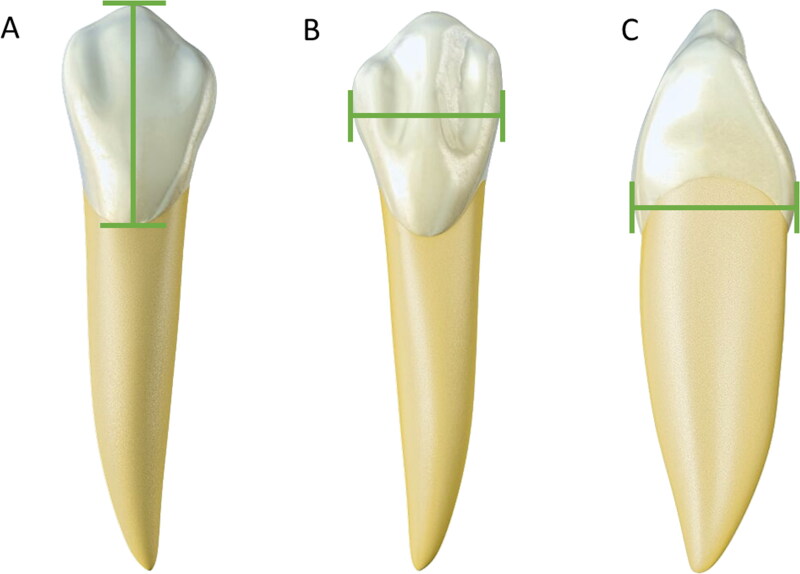
Metric analyses with statistical significance in sex estimation — canine teeth. (A) Cervical–incisal distance. (B) Mesiodistal width. (C) Vestibular–lingual distance. Males exhibit larger measures than females due to more voluminous teeth.

The measurement of mesiodistal and buccolingual distances (Figure 3) is most commonly used in ­sexual diagnosis [98]. Measurement values in males are higher than in females. Male teeth are more voluminous than female teeth. This premise applies to all teeth, but the upper and lower canines have the highest rate of dimorphism [92,99]. In young Indians (17–21 years old), a comparative study between mandibular canines showed a statistically significant difference in mesiodistal distance (6.5 mm in females *versus* 7.3 mm in males, *P* < 0.001) and intercanine distance (24.95 mm in males *versus* 26.07 mm in females, *P* < 0.001). Both measurements were reliable for estimating sex in this population [100].

The sexual dimorphism of teeth can also be attributed to genetic factors. The mineral phase of tooth enamel, the hardest and most resistant material in the human body, comprises amelogenin peptides [101]. The Y chromosome influences dental growth through amelogenin, a protein that participates in amelogenesis and dentinogenesis in males [102]. The average size of the dental crown on the X and Y chromosomes differs so that it is possible to differentiate sex by the paramount influence of amelogenin on the Y chromosome [103,104]. A recent study showed 100% accuracy in sex estimation using proteomics to identify amelogenin, whose peptide signal can remain stable for thousands of years, in a burial sample in central California [37].

On the X chromosome, however, amelogenin only acts in the amelogenesis process, promoting variation in the size of the teeth [102,104]. In this context, women’s maxillary canines possess thicker enamel in contrast with thicker dentine in males. These 3 D measurements (volumes and surface areas) were gathered to develop a discriminant function and resulted in 92.30% correct sex estimation in individuals from different geographic regions [91]. The amount of dentine in periapical radiographs can also guide sexual diagnosis in adolescent white Americans, being 6.5% on average greater in males’ maxillary permanent incisors than in females [105].

The incisor teeth also serve as a source of sexual diagnosis. In the Greek population, women showed more resemblance between the length of right and left incisors than men [99]. Female measurements are also statistically smaller for incisor teeth, with the cervical–incisal distance (crown length) being the most expressive, showing statistical significance (*P* = 0.001) for sex estimation in young Indians ­(19–23 years old) [88]. In agreement with this, a study in African Americans revealed that the mesiodistal crown dimension of central incisors was larger in males than in females [92]. Moreover, the cervical–incisal and mesiodistal distances of the upper central incisors vary according to the contour of the face of each person, corroborating with the individuality and facial harmony of the human being [106].

### Cervical vertebrae and hyoid bone

In the cervical vertebrae, the atypical atlas (C1) and axis (C2) vertebrae are the most useful in terms of sex estimation [107,108]. The atlas has a strong relationship with the base of the skull, and the male atlas supports a greater weight than the female atlas, given the greater muscle mass, bone density and brain weight in men. Consequently, the upper and lower articular faces, as well as the area of the vertebral foramen of the atlas, are more prominent in the male. A classic study used seven measures of these structures to construct regression equations and discriminating functions, with accuracies of 77%–85% and 75%–85%, respectively [107]. Likewise, a recent study in two European populations (Greek and Portuguese) revealed that the maximum height of the vertebral body (MHB) and the transversal diameter of the vertebral body (TDVB) of the cervical vertebrae showed accuracies of 80.3% and 84.5% in estimating sex [12].

The presence of the axis dens, its gradual process of fusion to the vertebral body, and growth at puberty make it a paramount element of sexual dimorphism in adult life. At puberty, bone elements begin to exhibit strong sexual dimorphism as a result of the action of oestrogen and testosterone. Additionally, the greater physical strength and muscle mass of male individuals impact bone landmarks, which are more prominent to accommodate the ligaments and muscles that perform more robust work [109–111].

**Figure 4.**
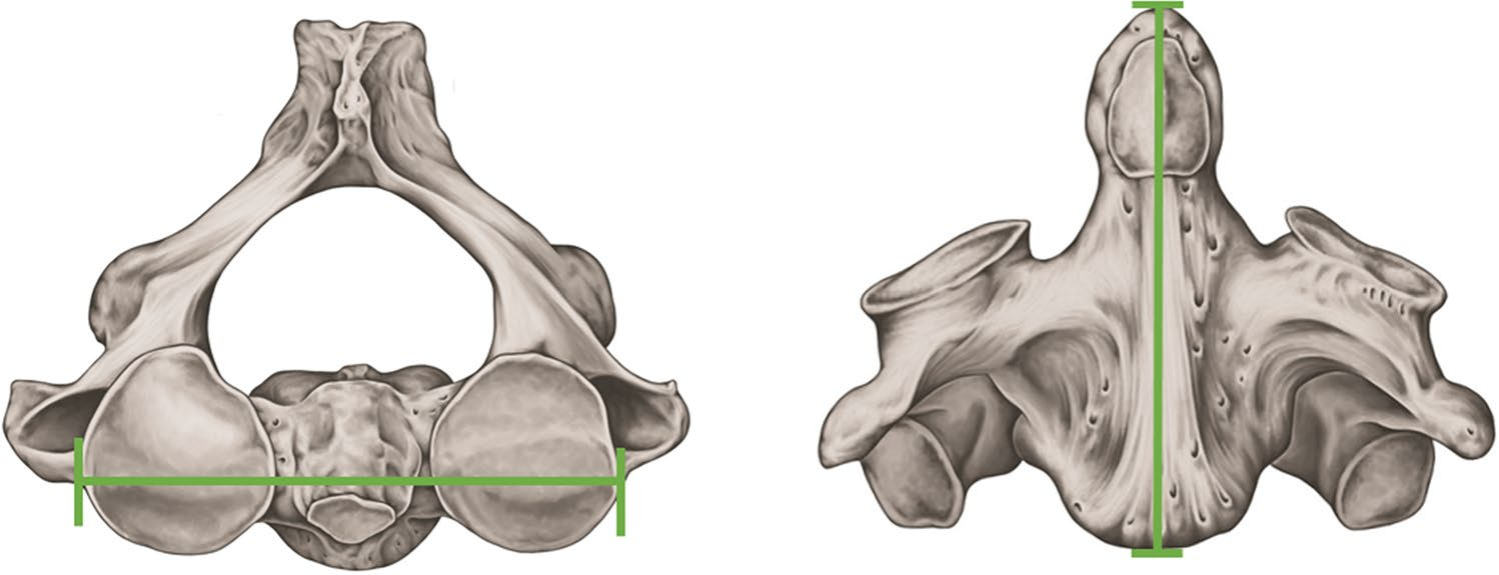
Metric analyses with statistical significance in sex estimation — axis. (A) Maximum amplitude between the upper articular faces. (B) Maximum sagittal length. Both measurements showed the highest discriminant values for sex estimation according to the Wescott method.

The application of the Wescott method to the collection of the Natural History Museum (London, UK) showed that the maximum sagittal length (MSL) and the maximum amplitude between the upper articular faces (MAUAF) of the axis (Figure 4) showed the highest discriminant value in the analysis between the sexes, reaching an accuracy of 83.3% [108]. The same method, when applied to axis samples from the United States population, showed an accuracy of 86.7% in estimating sex [112].

In the Portuguese population, the variables of MSL and MAUAF were again highly significant for sex estimation. However, this study tested, for the first time, the maximum width of the axis (MWA), which presented the strongest sexual dimorphism among the measures and should be applied in other populations [113]. In Brazil, a logistic regression model, using the length of the axis body (LAB), the length of the vertebral foramen (LVF), and the diameter of the transverse processes (DTP), demonstrated 72.4% accuracy in sexual estimation [114].

In the Japanese population, the analysis of CT images of the axis resulted in the construction of a five-variable discriminant function formula with 92.9% accuracy in estimating sex. This model, however, may not be applicable in other populations with different biotypes and nutritional patterns that impact on bone dimensions [115].

The hyoid bone has an average accuracy of between 72.1% and 92.3% in sex estimation when fusion is not complete. In completely fused hyoids, the methods are more accurate for men because male hyoids are larger. The methods applied for the sexual estimation of non-fused hyoids are more accurate in estimating sex in females because of their smaller size [116,117]. Ossification of the hyoid in women is completed 5 years before that of men [118,119].

The characterisation of sexual dimorphism in the hyoid usually relies on imaging examinations [120,121]. All measures used (width between the greater cornua, anteroposterior length, length of the greater cornua, body width, and distance between the lesser cornua) are significantly larger in men than in women [121]. In Japanese CT images, all measurements were more pronounced in men than in women. The perpendi­cular length from the most anterior point of the hyoid body to the line connecting the most distal points of the greater cornua and the linear distance between the two most lateral edges of the hyoid presented the most significant contribution to sexual diagnosis [115].

Bone density (higher in men) and the degree of partial bilateral fusion are also viable tools in estimating sex [122]. In the Turkish population, the use of 33 measurements in photographs of hyoid bones showed 92.5% accuracy for the estimation of males and 78.1% for females. Because of these controversies, the hyoid is not recommended for age estimation [123].

## Conclusion

The use of anatomical knowledge of bones and teeth combined with metric analyses can achieve high accuracy and low cost in sexual diagnosis. In the absence of the pelvic bone, the skull provides significant sexual dimorphism. The results outlined here highlight the importance of the glabellae, followed by the external frontal bone surface, the mandible, and the canine and incisor teeth, because isolated metric analyses of these structures can provide reliable sex estimation (accuracy > 80%). However, discriminant functions that consider multiple bone landmarks are attracting increased attention by virtue of their greater accuracy and lower interobserver error in estimating sex. These methods can benefit from the addition of cervical bone measurements, reaching an accuracy of >90% in sexual diagnosis.

In the forensic context, consideration should be given to recent migrations and lifestyle changes that have resulted in nutritional and hormonal modifications, affecting the bone structure. Thus, forensic anthropologists and scientists are often challenged to test new approaches and establish standards in different populations with different degrees of miscegenation to reach the greatest accuracy in the target population. More recently, integrated methods that use CT images or 3 D models coupled with anatomy-based metric analyses have shown enhanced accuracy, paving a promising new way to achieve reliable sex estimation.

## Authors’ contributions

Thamires Mello-Gentil and Vanessa Souza-Mello had the idea for the article, performed the literature search, data analysis, and drafted the manuscript and figures. Vanessa Souza-Mello critically revised the work. Both authors contributed and revised the final version of the manuscript.
